# Multi-omics reveals circadian regulation of bone homeostasis by gut microbiota metabolites: mechanisms and chronotherapeutic implications

**DOI:** 10.3389/fimmu.2025.1719445

**Published:** 2026-05-28

**Authors:** Mingdong Liu, Jiaqi Gong, Yaqi Liu, Jiayao Yu, Zepeng Hu, Zheng Liu

**Affiliations:** 1 Department of Orthopaedics, Affiliated Hospital of Shaoxing University, Shaoxing, Zhejiang, China; 2 Department of Pharmacology, School of Medicine, Shaoxing University, Shaoxing, Zhejiang, China; 3 Department of Nursing, School of Medicine, Shaoxing University, Shaoxing, Zhejiang, China

**Keywords:** gut microbiota, bone diseases, circadian rhythm, multi-omics, microbial therapeutics

## Abstract

The gut-bone axis plays a pivotal role in skeletal health, yet the integration of multi-omics approaches to elucidate circadian metabolite-bone interactions remains limited. This review synthesizes evidence from metagenomics, metabolomics, and germ-free models to uncover how microbiota-derived metabolites—including short-chain fatty acids (SCFAs), bile acids, tryptophan derivatives, and gaseous molecules—orchestrate bone remodeling in osteoporosis, osteoarthritis, and bone malignancies. Many studies demonstrate that SCFAs inhibit osteoclastogenesis via GPR43/HDAC signaling and promote osteoblast metabolic reprogramming, while bile acids enhance osteogenesis through FXR/Wnt/β-catenin activation. Tryptophan metabolites repair intestinal barrier integrity and modulate osteoimmunity via the AhR pathway. Single-cell omics reveal circadian oscillations of metabolite receptors (e.g., GPR43, FXR) in bone stromal cells, linking microbial diurnal rhythms to epigenetic regulation of bone turnover. We propose a novel “metabolite-immune-bone triad” model, highlighting microbiome-driven immunometabolic reprogramming as a central regulator of skeletal homeostasis. These insights advance precision microbial therapeutics and chrono-nutritional strategies, bridging multi-omics discoveries with clinical applications for bone disorders.

## 1 Introduction

As the global population ages, skeletal diseases such as osteoarthritis (OA) and osteoporosis (OP) have emerged as major public health challenges, collectively affecting over 550 million individuals worldwide (1, 2). These disorders are characterized by disrupted bone remodeling dynamics, yet current therapies targeting osteoblast-osteoclast equilibrium remain suboptimal due to incomplete mechanistic insights. Recent advances in the gut-bone axis (GBA) have revealed that gut microbiota-derived metabolites—including short-chain fatty acids (SCFAs), bile acids, and tryptophan derivatives—orchestrate bone homeostasis through immunometabolic crosstalk (3–5). Moreover, an imbalance in the gut microbiota may lead to bacterial and metabolite translocation, which can affect bone health (6–9). Although research in this field is still in its early stages, there is evidence to suggest that modulating the gut microbiota to improve bone health may be a promising therapeutic strategy.

Existing studies predominantly focus on static metabolite-bone interactions, neglecting the dynamic reciprocity between microbial circadian outputs and host bone cells. For instance, although SCFAs exhibit time-dependent effects on osteoclastogenesis, the mechanisms underlying circadian oscillations of G Protein-Coupled Receptor 43 (GPR43) in bone marrow stromal cells—and their disruption in OP—are yet to be elucidated (10). Similarly, bile acid-Farnesoid X Receptor (FXR) signaling shows phase-specific activity in osteoblasts (11), but how microbial metabolite rhythms align with skeletal transcriptional programs remains unknown. These gaps hinder the development of chronotherapeutic strategies targeting the GBA.

To address these challenges, this review underscores the transformative potential of multi-omics integration in elucidating the dynamic crosstalk between gut microbiota-derived metabolites and skeletal health. By leveraging metagenomics, metabolomics, and single-cell RNA sequencing (scRNA-seq), we delineate how microbial metabolites—such as SCFAs, bile acids, and tryptophan derivatives—orchestrate bone remodeling through receptor-mediated signaling (e.g., GPR43, FXR, AhR), epigenetic reprogramming, and immunometabolic regulation. Furthermore, we reveal circadian oscillations in metabolite receptor expression within bone stromal cells, linking microbial diurnal rhythms to osteoblast-osteoclast dynamics. These insights culminate in a novel “metabolite-immune-bone triad” model, which integrates circadian biology with microbiome-driven skeletal regulation. Our synthesis not only resolves prior knowledge gaps regarding temporal host-microbe interactions but also provides a mechanistic foundation for chronotherapeutic and nutritional interventions aimed at treating bone disorders. This work sets the stage for future translational studies, enabling precision medicine approaches that harness microbial metabolites for skeletal disease management.

## 2 Bone homeostasis and the role of GBA in bone diseases

### 2.1 Fundamentals of bone homeostasis

The skeleton is a dynamic organ that maintains its strength through continuous remodeling, a process that relies on the delicate balance between bone formation mediated by osteoblasts and bone resorption mediated by osteoclasts. Osteoblasts originate from mesenchymal stem cells, and their differentiation is driven by key signaling pathways such as Wnt/β-catenin and bone morphogenetic proteins; osteoclasts, on the other hand, derive from hematopoietic stem cells, and their formation is centrally regulated by the RANKL/RANK/OPG system. Osteocytes embedded in the bone matrix act as central regulators, coordinating the activities of the aforementioned two types of cells by sensing mechanical signals and secreting factors such as sclerostin (which inhibits the Wnt pathway) and RANKL. Additionally, mesenchymal stem cells in the bone marrow microenvironment compete between osteogenic and adipogenic differentiation pathways, and the tilt of this balance (i.e., the accumulation of bone marrow adipose tissue) is an important mechanism driving bone loss. Finally, systemic hormones such as estrogen play a key role in maintaining bone homeostasis by inhibiting osteoclastogenesis and promoting osteogenesis. Understanding this fundamental framework is crucial for exploring how gut microbiota and their metabolites remotely regulate this complex network through immune, metabolic, and endocrine pathways.

### 2.2 The role of GBA in bone diseases

Recent advances in microbiology and skeletal disease research have brought increasing attention to the emerging concept of the GBA. This concept delineates a bidirectional communication system wherein gut microbiota influence bone metabolism and health through diverse pathways. Specifically, gut microbes modulate bone density, formation, and resorption. The complex mechanisms underlying the GBA include the production of SCFAs, modulation of the immune system, and direct or indirect effects of gut microbial metabolites on bone cells (5, 12, 13).

OP is a disease characterized by reduced bone density and altered microstructure, which significantly increases the risk of fractures. Studies have shown that gut microbes can regulate bone density by affecting vitamin D synthesis and calcium absorption (5, 8, 14), and SCFAs (such as butyrate and propionate) produced by gut microbes can stimulate the activity of osteoblasts and inhibit the formation of osteoclasts, thereby helping to maintain and enhance bone mass (15–19). However, an imbalance in gut microbes can trigger chronic low-grade inflammation, which is associated with the development of OP (5, 20–22). Inflammatory cytokines such as TNF-α and IL-6 can enhance osteoclast activity, leading to bone loss. Some studies found that modulating the gut microbiota can improve bone density, further confirming the potential regulatory role of gut microbes in OP (23, 24).

OA, characterized by articular cartilage degradation and joint inflammation, may be influenced in its development by gut microbiota through the gut-joint axis. Research indicates that lipopolysaccharides (LPS) derived from gut microbes can induce systemic inflammation, thereby exacerbating joint inflammation and accelerating disease progression (25, 26). Furthermore, alterations in gut microbiota composition can modulate immune responses. For instance, probiotics may alleviate OA symptoms by regulating T-cell activity (5, 27).

**Figure 1:**
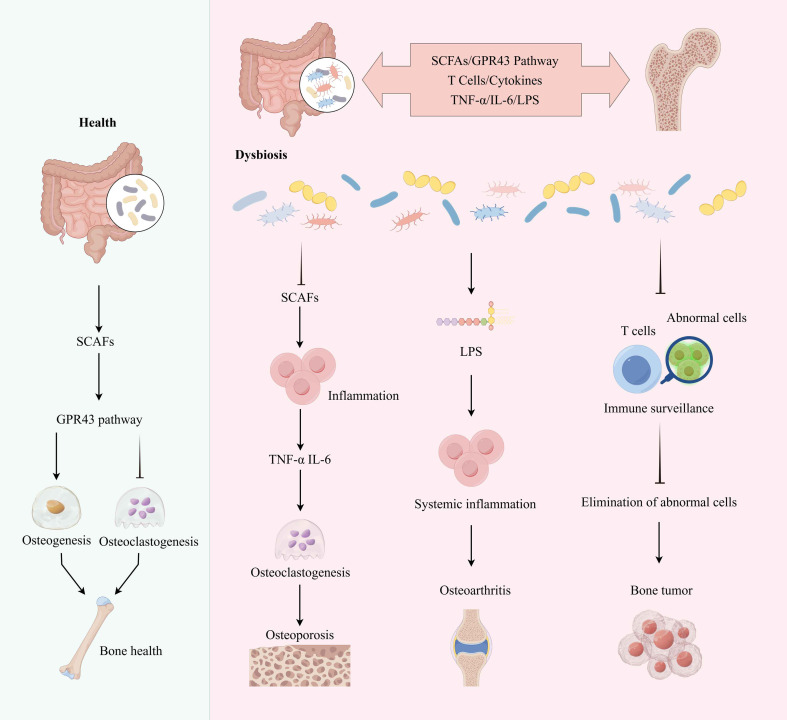
A panoramic view of the regulatory mechanisms of the microbiota-gut-bone axis in skeletal diseases. Under healthy conditions (left side), short-chain fatty acids (SCFAs, e.g., butyrate/propionate) produced by the gut microbiota activate the GPR43 signaling pathway, directly promoting osteogenesis and inhibiting osteoclastogenesis to maintain bone health. Under gut dysbiosis conditions (right side), decreased SCFA production and microbial imbalance drive skeletal pathogenesis through three distinct pathways: (1) Osteoporosis: Reduced SCFAs and increased pro-inflammatory cytokines (e.g., TNF-α/IL-6) disrupt the bone formation/resorption balance, favoring bone loss; (2) Osteoarthritis: Lipopolysaccharide (LPS) triggers systemic inflammation that spreads to joints, promoting local inflammation and cartilage degradation; (3) Bone Tumor: Dysbiosis impairs immune surveillance (e.g., T cell dysfunction), allowing abnormal cell proliferation and bone tumor formation due to failed elimination. ⊥: Inhibits/decreases.

The development of bone tumors is intricately linked to chronic inflammation, immune dysregulation, and gut microbiota dysbiosis through GBA. Emerging evidence indicates that gut microbiota-derived metabolites modulate bone tumorigenesis and progression through multiple mechanisms. Dysbiosis-driven systemic inflammation (e.g., elevated circulating LPS) and impaired immune surveillance disrupt bone microenvironment homeostasis, facilitating tumor cell survival and osteolytic activity. Experimental studies demonstrate that gut microbiota depletion reduces bone metastasis burden in murine models, while fecal microbiota transplantation (FMT) from tumor-bearing hosts accelerates osteosarcoma growth, underscoring microbiota’s causal role in bone malignancy (28, 29) (**Figure 1**).

The gut microbiota modulates bone tumors through three main pathways. First, microbial metabolites such as SCFAs, notably butyrate, exert direct anti-tumor effects by inhibiting histone deacetylases (HDACs) in osteosarcoma cells, promoting cell cycle arrest and apoptosis via p21/WAF1 upregulation. Butyrate also enhances CD8+ T-cell infiltration and polarizes tumor-associated macrophages (TAMs) toward anti-tumor M1 phenotypes via GPR43 signaling (30, 31). Clinically, reduced fecal SCFAs correlate with accelerated bone tumor progression (32, 33). Second, dysbiosis impairs immune surveillance by reducing IL-17A+ γδ T cells and NK cell activity, hindering the elimination of metastatic cells in bone marrow, as shown in germ-free models (34, 35). Tryptophan metabolites (e.g., indole-3-aldehyde) activate the aryl hydrocarbon receptor (AhR) in dendritic cells, boosting anti-tumor CD8+ T-cell responses (36). Third, gut barrier disruption enables pathobiont translocation (e.g., Fusobacterium nucleatum), increasing systemic LPS. This activates TLR4/NF-κB signaling in bone stromal cells, elevating IL-6 and TNF-α, which enhance osteoclastogenesis and form a pre-metastatic niche (37, 38).

## 3 Role of microbiota metabolite on the cell metabolsim of bone diseases

### 3.1 Gut microbiota and bone metabolism

The gut microbiota, a complex ecosystem of trillions of microorganisms, significantly influences bone metabolism. Studies demonstrate that probiotics (e.g., Lactobacillus and Bifidobacterium) can enhance bone density (39), while opportunistic pathogens (e.g., Enterobacteriaceae) are linked to bone loss (23). In individuals and models with a conventional gut microbiota, reduced microbial diversity has been observed to correlate with decreased bone density. Notably, the relationship between microbial presence and bone mass is complex and non-linear. Germ-free (GF) mice, which entirely lack microbiota, exhibit a unique skeletal phenotype characterized by suppressed osteoclastogenesis and increased trabecular bone volume compared to conventionally raised mice. This apparent paradox is attributed to the underdeveloped immune system in GF mice, particularly the reduction in osteoclast precursors and pro-osteoclastic immune cells (e.g., CD4+ T cells), leading to diminished bone resorption. This highlights that the complete absence of microbiota creates a distinct immunometabolic state not directly comparable to simply having ‘low diversity’ within a conventional microbiome (23). Additionally, gut microbiota indirectly regulate bone metabolism through immune modulation and inflammatory responses. For example, OP patients exhibit diminished gut microbiota diversity alongside reduced bone density.

A key mechanism involves SCFAs—microbial metabolites derived from dietary fiber fermentation. SCFAs (e.g., acetate, butyrate) mediate bone homeostasis through epigenetic regulation (e.g., histone deacetylase inhibition) and receptor signaling (e.g., GPR activation). Their roles in osteoblast promotion, osteoclast suppression, and circadian synchronization of bone remodeling are detailed in Section 3.2 (14, 17, 40, 41).

### 3.2 Metabolite signaling pathways in bone homeostasis and diseases

#### 3.2.1 Bidirectional regulation by SCFAs

**Figure 2:**
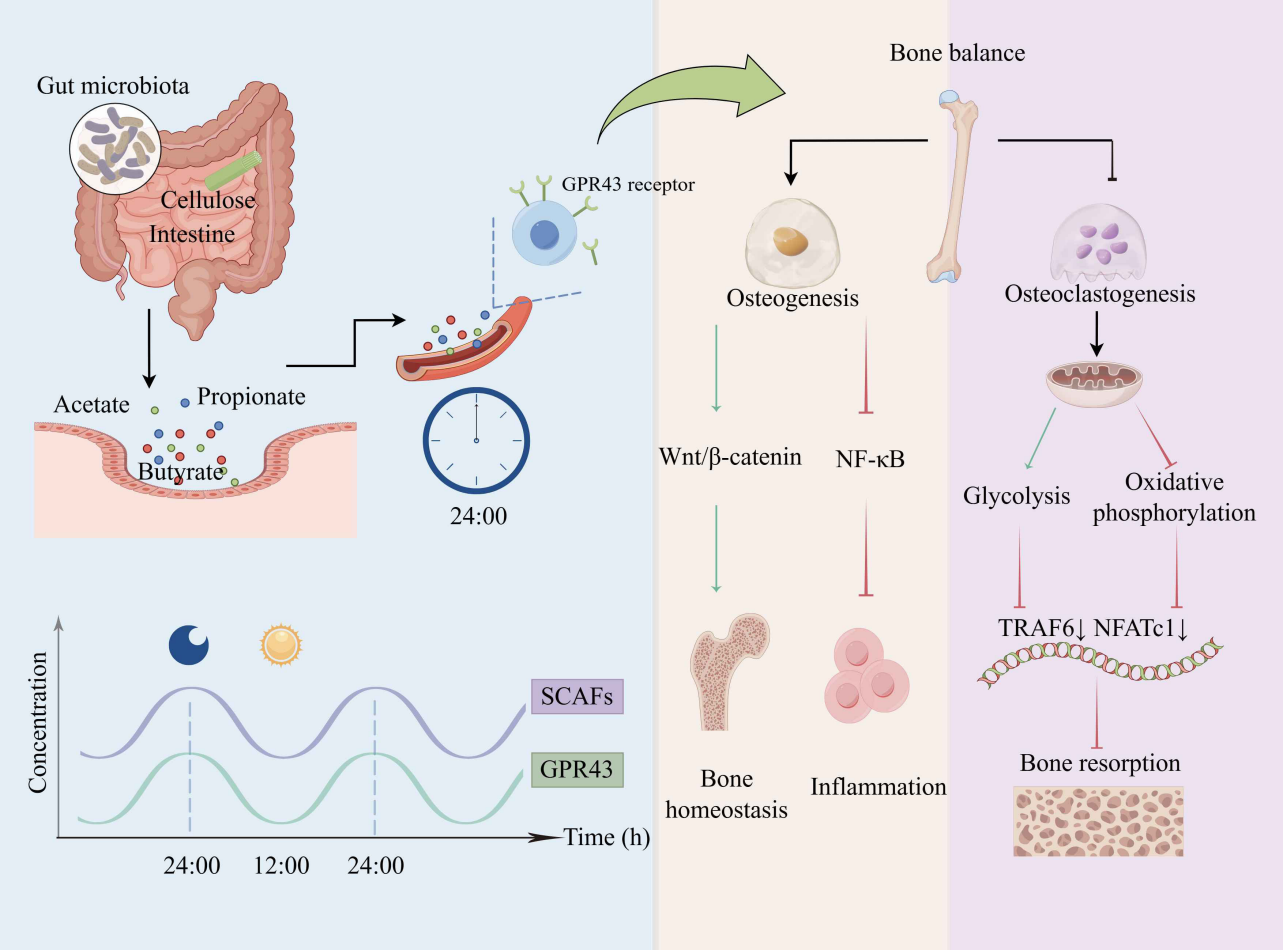
Circadian regulation of bone metabolism by gut microbiota-derived short-chain fatty acids (SCFAs) via GPR43 signaling. The gut microbiota ferments dietary cellulose to produce SCFAs (acetate, propionate, butyrate), which exhibit diurnal fluctuations in concentration. SCFAs activate the rhythmically expressed GPR43 (G protein-coupled receptor 43) in bone cells, peaking at midnight. This signaling cascade promotes osteogenesis by enhancing the Wnt/β-catenin pathway and suppressing NF-κB-mediated inflammation. Concurrently, SCFAs drive metabolic reprogramming in bone cells, balancing glycolysis and oxidative phosphorylation to favor bone mineralization over resorption. Ultimately, the gut microbiota–SCFA–GPR43 axis orchestrates bone homeostasis through integrated circadian, metabolic, and immune mechanisms. ⊥: Inhibits/decreases.

SCFAs are primary products of gut microbial fiber fermentation. Absorbed via the colon and transported systemically, they serve as energy substrates or signaling molecules. Acetate circulates widely, while propionate is liver-metabolized (17, 42–44). SCFAs exert their effects on bone metabolism through multiple interconnected mechanisms. SCFAs activate GPR41, GPR43, GPR109A and free fatty acid receptors (FFAR2/3), initiating intracellular signaling pathways that regulate hormone secretion, inflammation, and immune function. These receptors exhibit rhythmic expression patterns in bone marrow stromal cells, with GPR43 peaking at midnight, aligning with circadian metabolite flux and suggesting a role for SCFAs in synchronizing bone remodeling through temporal regulation (17, 45). Furthermore, butyrate and propionate drive metabolic reprogramming in osteoclasts by shifting their energy metabolism toward glycolysis and away from oxidative phosphorylation. This metabolic shift downregulates osteoclastogenesis-related genes such as TRAF6 and NFATc1, thereby suppressing bone resorption and enhancing bone density (14, 17). Epigenetically, SCFAs modulate histone acetylation (e.g., H3K27ac) by inhibiting HDACs, influencing osteoblast differentiation and osteoclast activity. For instance, butyrate directly targets TRAF6 and NFATc1 expression to maintain bone homeostasis (40, 41). Advances in multi-omics approaches, including single-cell transcriptomics (scRNA-seq) and metabolomics, have uncovered circadian oscillations of metabolite receptors (e.g., GPR43, FXR) in osteoprogenitor cells. These findings provide a framework for understanding how microbial metabolites like SCFAs synchronize bone remodeling through dynamic interactions between epigenetic modifications (e.g., histone acetylation) and metabolic pathways, as evidenced by temporal alignment of receptor expression and metabolite flux (17) (**Figure 2**).

Although low concentrations of SCFAs (especially butyrate) have been shown to promote osteoblast differentiation and bone formation, *in vitro* experiments have clearly demonstrated that higher concentrations of butyrate can inhibit the proliferation and differentiation of osteoblasts. The underlying mechanism is related to the induction of cell cycle arrest. For example, studies have indicated that histone deacetylase inhibitors (HDACi) such as butyrate and trichostatin A (TSA), at concentrations that can cause high levels of histone acetylation, will inhibit the expression of osteoblast marker genes (such as Osteocalcin) and matrix mineralization. The mechanism is that HDACi upregulate the expression of the cell cycle-dependent kinase inhibitor p21 Cip1/WAF1, leading to cell cycle arrest of osteoblast precursors in the G1 phase and blocking their differentiation into mature osteoblasts (46). This suggests that the bidirectional regulation of osteoblasts by SCFAs follows the “hormesis” effect (beneficial at low doses, harmful at high doses). Moreover, under specific pathological conditions, SCFAs may exacerbate inflammation and produce complex effects through different immune pathways in specific microenvironments (such as rheumatoid arthritis synovium). For example, studies have shown that SCFAs can promote the differentiation of Th17 cells, which are key subsets of cells that promote inflammation and bone erosion, potentially leading to adverse consequences in autoimmune bone diseases (47).

#### 3.2.2 Bile acid-FXR/Wnt cascade

Bile acids, synthesized by the liver, are essential for the digestion and absorption of lipids and are reabsorbed in the enterohepatic circulation. They also influence bone metabolism through the FXR, which is highly expressed in osteoblasts and plays a significant role in their proliferation (48, 49) FXR, as a major bile acid receptor and a member of the nuclear receptor superfamily, plays a central role in maintaining metabolic homeostasis. It was initially widely recognized for its key roles in regulating bile acid synthesis, lipid metabolism, and glucose homeostasis in the liver and intestine. Upon binding of bile acids to FXR, a series of downstream events are triggered, including the induction of small heterodimer partner (SHP) expression, which in turn inhibits bile acid synthesis and regulates their enterohepatic circulation. In recent years, studies have revealed that FXR also has important functions in bone metabolism. FXR is highly expressed in osteoblasts, and its activation has been shown to directly regulate the expression of osteogenesis-related genes and affect bone energy metabolism, thereby closely linking bile acid signaling to the process of bone formation. Studies have shown that geniposidic acid (GPA) can activate the FXR signaling pathway, upregulating the mRNA expression of osteogenic markers such as Runx2, Osx, and Alpl, increasing the levels of related proteins and Alkaline phosphatase (ALP) enzyme activity, and promoting osteoblast mineralization (50). In an ovariectomized mouse model, GPA was able to increase bone density and quality, enhance bone formation rates, and elevate serum bone formation marker levels, as well as increase the expression of factors such as FXR, SHP, and RUNX2. In FXR gene-knockout mice, the effects of GPA were diminished, confirming that it promotes bone formation through the FXR/RUNX2 signaling pathway, offering a new strategy for the treatment of OP (5, 6, 51, 52).

Furthermore, bile acids enhance osteoblast function through the Wnt/β-catenin signaling pathway, which is crucial for the differentiation of bone marrow mesenchymal stem cells (BM-MSCs), osteoblast formation and function, osteoclast differentiation, and bone resorption. Wnt signaling promotes the differentiation of BM-MSCs into osteoblasts and inhibits adipocyte differentiation, such as Wnt3a activating Transcriptional coactivator with PDZ-binding motif (TAZ) to promote osteogenic differentiation (8, 53). It also enhances osteogenic differentiation and inhibits adipogenic differentiation through the noncanonical JNK pathway. In osteoblasts, Wnt signaling activates Runx2 through β-catenin/TCF1, promoting differentiation and bone formation, while also increasing the expression of bone morphogenetic proteins, which promote osteoblast proliferation and differentiation (54–56). Wnt signaling also inhibits osteoclast differentiation by regulating the expression of RANKL and OPG, such as activating Wnt signaling in osteoblasts to suppress RANKL expression and increase OPG expression, thereby inhibiting bone resorption (57, 58). The activation of the Wnt signaling pathway enhances the sensitivity of osteocytes to mechanical stimuli, promoting bone formation and inhibiting bone resorption. Abnormalities in the Wnt signaling pathway are associated with various bone diseases, such as the inhibition of Wnt signaling leading to bone loss in OP and abnormal activation leading to increased bone mass in osteomalacia (54, 59).The regulation of the Wnt signaling pathway provides new targets for the treatment of bone diseases (57).

**Figure 3:**
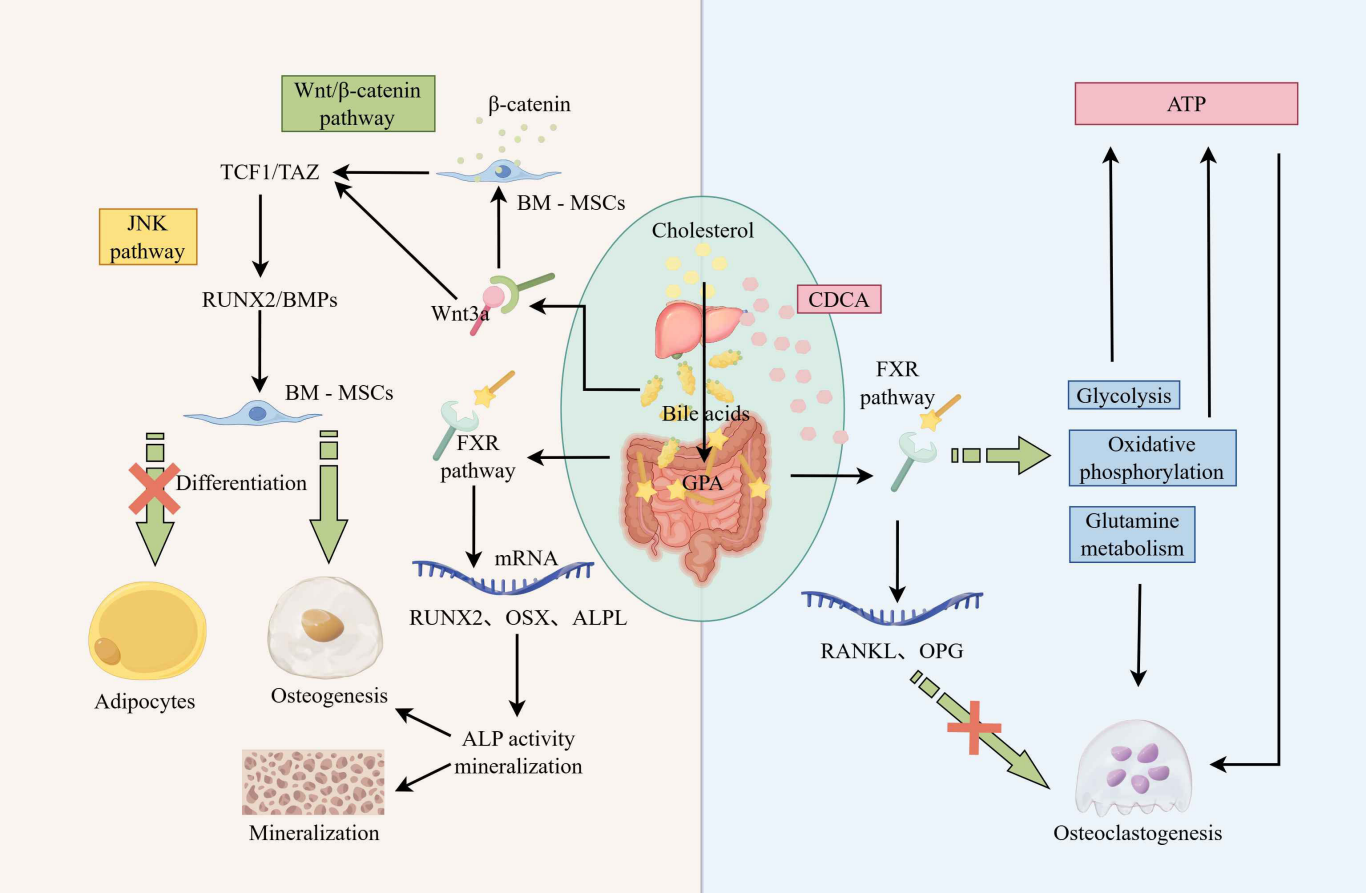
Regulation of bone metabolism by bile acids through FXR and Wnt/β-catenin signaling pathways. Bile acids (e.g., CDCA) and synthetic agonists like GPA activate the Farnesoid X receptor (FXR) pathway. This upregulates the expression of RUNX2/OSX/ALPL, enhancing alkaline phosphatase activity and mineralization. Concurrently, bile acids enhance Wnt/β-catenin signaling via TCF1/TAZ and the non-canonical JNK pathway, further driving BM-MSCs toward osteogenesis and inhibiting adipogenesis. Downstream effects include the inhibition of osteoclastogenesis through metabolic reprogramming (glycolysis, oxidative phosphorylation, and glutamine metabolism). Overall, the FXR and Wnt pathways integrate to coordinate bone formation and resorption.

Bile acids can inhibit the formation and activity of osteoclasts. For example, chenodeoxycholic acid (CDCA) inhibits osteoclast differentiation through the FXR signaling pathway (48, 60, 61). The mechanism may be related to the upregulation of osteoprotegerin and other osteoclastogenesis inhibitory factors in osteoblasts after FXR activation, thereby indirectly inhibiting osteoclast formation. In addition, studies have shown that the activation of FXR can change the intracellular metabolic environment, but this mainly occurs in osteoblasts and other stromal cells, where it dominates the bone microenvironment by promoting anabolic metabolism, ultimately exerting a net inhibitory effect on osteoclasts (62, 63). In summary, current evidence indicates that bile acids mainly exert an inhibitory effect on osteoclastogenesis in the bone microenvironment through the FXR signaling pathway (**Figure 3**).

#### 3.2.3 Tryptophan metabolite-AhR axis

**Figure 4:**
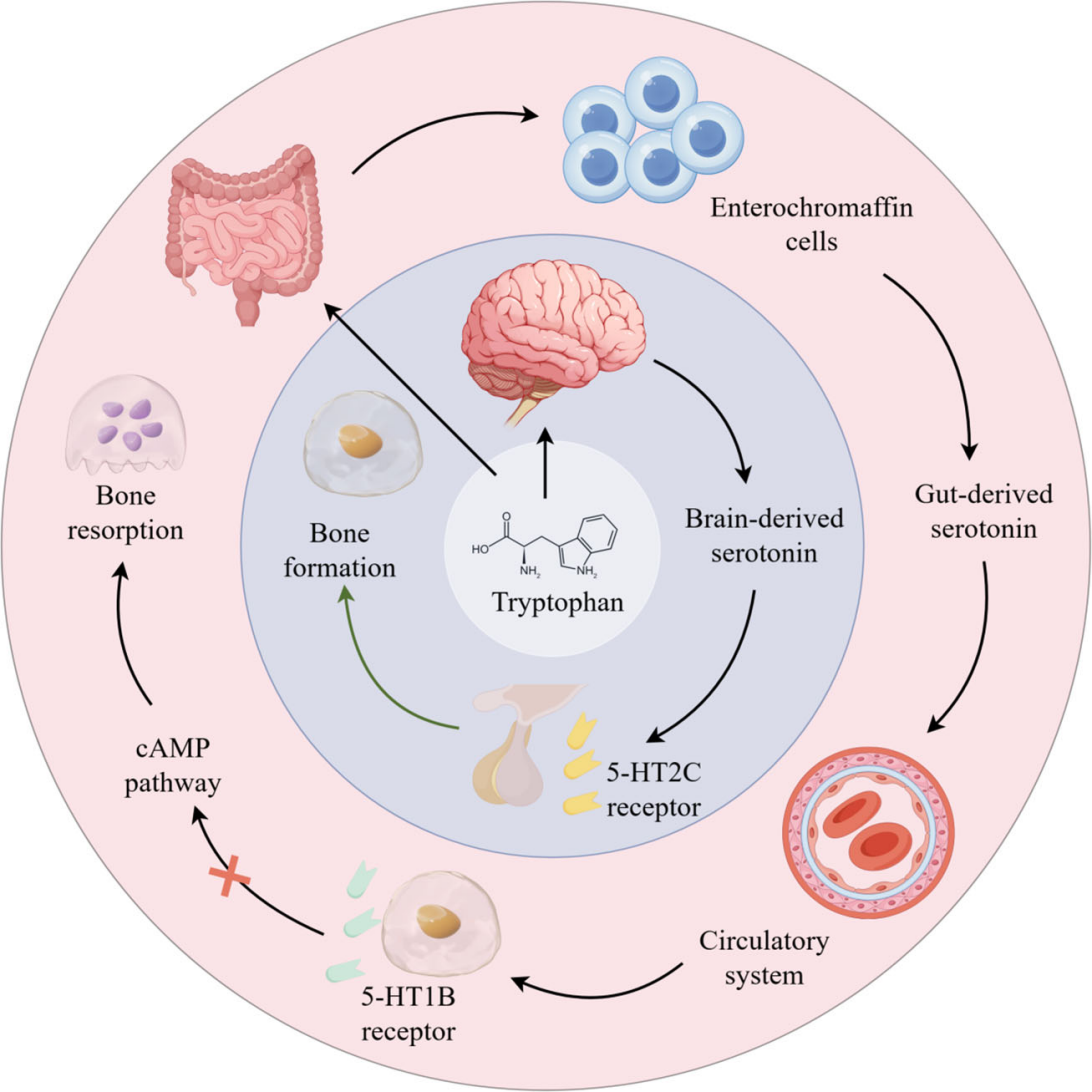
Opposing effects of gut- and brain-derived serotonin on bone remodeling. Tryptophan metabolism produces serotonin in two distinct compartments with opposing effects on bone. Gut-derived serotonin (95% of total) acts on osteoblasts via the 5-HT1B receptor, inhibiting bone formation by suppressing cAMP signaling. In contrast, brain-derived serotonin signals through hypothalamic 5-HT2C receptors to promote bone formation and reduce resorption. This duality explains the bone loss associated with drugs that increase peripheral serotonin (e.g., SSRIs), highlighting the gut-derived pathway as a therapeutic target.

Tryptophan, a precursor to serotonin (5-hydroxytryptamine), melatonin, kynurenine, and niacin, has metabolic byproducts that influence bone remodeling. In diseases of bone metabolism, changes in tryptophan levels occur and play a role in osteoblast differentiation (64) Gut-derived serotonin, which accounts for approximately 95% of the body’s total serotonin, is synthesized by enterochromaffin cells and does not cross the blood-brain barrier. It acts as a potent inhibitor of bone formation. Mechanistically, gut-derived serotonin binds to the 5-HT1B receptor on osteoblasts, inhibiting their proliferation by suppressing cyclic AMP (cAMP) production and cyclin expression. In contrast, brain-derived serotonin, synthesized in the raphe nuclei of the brainstem, functions as a positive regulator of bone mass by enhancing bone formation and reducing bone resorption through a sympathetic nervous system-independent pathway, primarily involving the 5-HT2C receptor in the hypothalamus. The dual origin and opposing actions of serotonin create a complex therapeutic landscape, especially in the context of the widely used selective serotonin reuptake inhibitors (SSRIs). SSRIs increase synaptic serotonin levels to treat depression but also elevate peripheral serotonin bioavailability. Epidemiological studies consistently report that long-term SSRI use is associated with reduced bone mineral density (BMD) and an increased risk of fractures, an effect attributed to the unopposed inhibitory action of gut-derived serotonin on osteoblasts. This presents a clinical dilemma: the neuropsychiatric benefits of SSRIs may come at the cost of skeletal health, particularly in vulnerable populations such as postmenopausal women. Consequently, selectively inhibiting gut-derived serotonin synthesis without affecting the central nervous system has emerged as a promising therapeutic avenue for bone anabolism. Additionally, microbial tryptophan metabolites indole-3-acetic acid (IAA) and indole-3-propionic acid can significantly improve bone loss by repairing intestinal barrier integrity (64–67). These metabolites activate the aryl hydrocarbon receptor (AhR) in the gut, stimulate the Wnt/β-catenin signaling pathway, repair the intestinal barrier, promote osteoblastogenesis, and inhibit osteoclasts. Tryptophan metabolites affect the senescence of musculoskeletal stem cells, with metabolites of the kynurenine pathway such as 3-hydroxykynurenine, kynurenic acid, and anthranilic acid promoting bone aging, reducing bone mineral density, and increasing fracture risk (68). In contrast, 3-hydroxyanthranilic acid, xanthuric acid, picolinic acid, quinolinic acid, and NAD+ increase bone mineral density and reduce fracture risk (69–72) (**Figure 4**).

#### 3.2.4 Gaseous microbiota-derived metabolites

Emerging evidence indicates that gaseous metabolites derived from gut microbial metabolism of dietary fiber, sulfur-containing amino acids, and proteins—including hydrogen sulfide (H_2_S), nitric oxide (NO), ammonia (NH_3_), carbon monoxide (CO), and others—function not as waste products but as signaling molecules that regulate skeletal homeostasis via the gut–bone axis (GBA). Among these, H_2_S acts as a protective gaseous mediator, similar to NO and CO, and exerts significant effects on nervous, cardiovascular, and skeletal systems, as well as inflammatory processes. It supports skeletal development through antioxidant, anti-inflammatory, and anti-apoptotic mechanisms, notably by rebalancing bone remodeling in OP, stimulating bone formation, and preserving skeletal integrity (73). Please note that CO is different from carbon dioxide and is a gaseous molecule that is often considered toxic due to its high affinity for hemoglobin at high concentrations. However, at low concentrations that are physiologically relevant, it exerts anti-inflammatory and reparative effects via the HO-1 pathway. Meanwhile, gut-derived serotonin, which is regulated by the microbiota, inhibits bone formation when upregulated, illustrating the complex gut-brain-bone crosstalk.

Produced by sulfate-reducing bacteria such as Desulfovibrio spp., H_2_S promotes osteoblast differentiation via the Wnt/β-catenin pathway and inhibits inflammation-induced osteoclastogenesis through suppression of NF-κB signaling. Studies using the H_2_S donor sodium hydrosulfide (NaHS) have shown attenuation of dexamethasone (Dex)-induced reactive oxygen species (ROS) generation and ATP depletion, mediated via AMPK pathway activation (74, 75). Furthermore, H_2_S protects osteoblasts from Dex-induced apoptosis and necrosis through AMPK-dependent mechanisms, highlighting its cytoprotective and antioxidant roles (76). Notably, H_2_S-releasing compounds have been shown to increase bone mineral density in murine OP models.

NO, generated via microbial nitrite reduction and host eNOS activity, bidirectionally modulates osteoclast–osteoblast coupling through the cGMP–PKG pathway. Bone-implant materials engineered for NO release have demonstrated efficacy in enhancing defect repair. Elevated NH_3_, produced by proteolytic microbes, aggravates myeloma-related osteolysis by stimulating osteoclast activity, spurring interest in targeting ammoniagenic pathways therapeutically. CO, via the HO-1 pathway, exerts anti-inflammatory and reparative effects, while gut-derived serotonin—under microbiota control—inhibits bone formation when upregulated, illustrating complex gut–brain–bone crosstalk.

**Figure 5:**
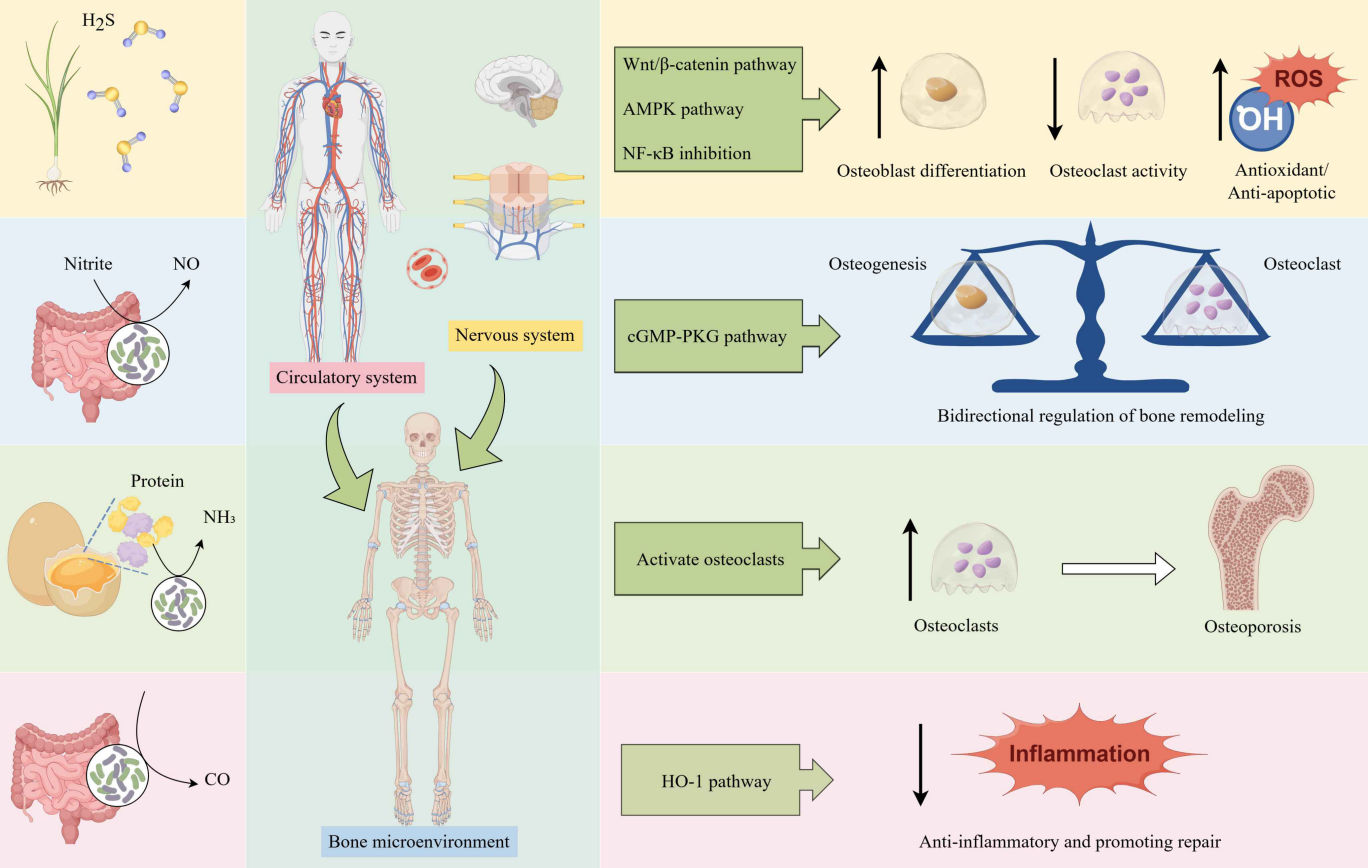
Mechanisms of gut microbiota-derived gaseous metabolites in bone remodeling. Gut microbial fermentation produces gaseous metabolites that enter circulation and regulate bone homeostasis. H_2_S (e.g., from Desulfovibrio) promotes osteogenesis via Wnt/β-catenin signaling and inhibits osteoclastogenesis via NF-κB suppression and AMPK-mediated antioxidant effects. NO bidirectionally modulates osteoclast-osteoblast coupling through the cGMP-PKG pathway. NH_3_ (from proteolytic bacteria) activates osteoclasts and exacerbates bone resorption. CO exerts anti-inflammatory and pro-repair effects via the HO-1 pathway. These mechanisms inform targeted gas-delivery systems (e.g., nano-scaffolds) for treating bone defects and osteoporosis. Abbreviations: AMPK, AMP-activated protein kinase; HO-1, heme oxygenase-1; NF-κB, nuclear factor kappa-light-chain-enhancer of activated B cells.↑: Promotes/increases; ↓: Inhibits/decreases.

Capitalizing on these mechanisms, advanced micro- and nano-delivery systems enabling controlled release of H_2_S, NO, and CO are being developed. These innovations hold translational promise for preventing implant-related infections, enhancing bone regeneration, and enabling combinatorial gas therapies for complex skeletal disorders (**Figure 5**).

### 3.3 Immune regulatory network

#### 3.3.1 Microbiota-immune cell interactions

Modulating the gut microbiota has emerged as a potential therapeutic strategy for rheumatoid arthritis (RA), where probiotics (e.g., Lactobacillus) and prebiotics (e.g., dietary fiber) alleviate RA symptoms and slow disease progression by improving microbial composition (77). For instance, Lactobacillus species prevent bone loss and maintain bone density through modulating the Treg-Th17 cell ratio and inhibiting osteoclastogenesis (78, 79). Additionally, polyphenolic compounds such as ellagic acid exhibit anti-inflammatory effects by regulating the gut microbiota (80, 81). Beyond RA, the gut microbiota critically influences bone remodeling by balancing Th17/Treg dynamics (82). Probiotics (e.g., Lactobacillus reuteri) suppress osteoclast precursor (CD11b+/Gr1-) recruitment and reduce pro-inflammatory cytokines (TNF-α, IL-6) via Treg cell expansion (83, 84). Single-cell immunogenomics further reveal enhanced Th17 infiltration and metabolic reprogramming of GPR43+ macrophages in osteoporotic bone marrow, highlighting microbial metabolites’ role in mediating “metabolite-immune dialogue” to regulate the bone microenvironment (85).

#### 3.3.2 Bone loss mediated by inflammatory cytokines

Microbial products such as LPS induce chronic low-grade inflammation and promote RANKL-dependent osteoclastogenesis through the TLR4/NF-κB pathway (86, 87). Clinical cohort data show that serum TNF-α levels in OA patients are negatively correlated with gut microbiota diversity (88). Mechanistically, SCFAs produced by gut microbiota can reduce NLRP3 inflammasome activation by inhibiting histone deacetylase 6 (HDAC6), thereby attenuating inflammatory responses (89, 90). Additionally, FMT therapy has been shown to reduce IL-1β levels in a randomized controlled trial (RCT), verifying the clinical translational potential of the “immune-bone axis.” These findings highlight the critical role of the gut microbiota in modulating systemic inflammation and its impact on bone health, suggesting that microbiota-targeted therapies may have therapeutic potential for inflammatory bone diseases (49, 90–92).

#### 3.3.3 Microbiota-immune clock synergy

Disruption of circadian rhythms alters the structure and function of the gut microbiota, impacting host metabolism and immune function (93–95). Microbial metabolites (e.g., SCFAs) regulate host immune cell function and infiltration by activating receptors (e.g., GPR43) (96, 97) and modulate gene expression via epigenetic mechanisms like histone modifications (96, 98). These actions affect bone matrix cells, thereby regulating bone metabolism (18, 99, 100). Concurrently, circadian rhythms directly regulate immune cell function; their disruption impairs intestinal immune barrier function, increasing sensitivity to endotoxins like LPS (101–103). Therefore, the gut microbiota, through its metabolic signaling and in coordination with the host’s immune circadian rhythms, constitutes the “metabolite-immune-bone triad model” to maintain bone homeostasis (5, 49, 104).

Experimental validation of the “metabolite-immune-bone triad” in animal models provides mechanistic credibility to this integrative framework: Probiotic-mediated Treg expansion ameliorates bone loss in ovariectomized (Ovx) mice, where Lactobacillus reuteri supplementation increased bone marrow Treg frequency by 2.1-fold, suppressed osteoclast precursor recruitment, and reduced trabecular bone loss by 32% compared to controls. This correlated with decreased serum TNF-α and IL-6 levels, confirming microbiota-immune crosstalk in skeletal protection. Germ-free (GF) mouse studies reveal direct immune-bone coupling: GF mice exhibit 40% higher trabecular bone volume alongside reduced CD4+T cells and osteoclast precursors (CD11b+/Gr1-) in bone marrow.Reconstitution with conventional microbiota normalized immune cell profiles and decreasing the bone mass to normal levels. SCFAs synchronize circadian bone remodeling through epigenetic-immune coordination. Butyrate gavage in mice elevated H3K27ac acetylation at GPR43 promoter regions in bone stromal cells, amplifying midnight receptor expression by 3.5-fold. This enhanced Treg differentiation and suppressed NLRP3 inflammasome activation, reducing osteoclastogenesis by 45% in circadian-misaligned models.These findings solidify the triad model: microbial metabolites (e.g., SCFAs) rhythmically engage immune cells (Treg/Th17) and epigenetic machinery (H3K27ac) to orchestrate bone remodeling, offering actionable targets for chronotherapeutic interventions.

### 3.4 Therapeutic implications: translating mechanistic insights into interventions

The comprehensive mechanistic studies reviewed above reveal that gut microbiota-derived metabolites (SCFAs, bile acids, tryptophan derivatives, and H_2_S) precisely regulate bone homeostasis through multidimensional pathways: receptor signaling (e.g., GPR43, FXR, AhR), epigenetic reprogramming (HDAC inhibition/H3K27ac acetylation), metabolic remodeling (glycolysis-oxidative phosphorylation balance), immune modulation (Th17/Treg balance, inflammatory cytokine inhibition), and circadian coordination (receptor oscillatory expression). These findings lay a direct theoretical foundation and provide actionable targets for developing precision therapies targeting the gut-bone axis:

SCFAs pathway inhibits osteoclast differentiation and promotes osteoblast metabolic reprogramming through GPR43/HDAC signaling, directly supporting the use of SCFA-producing probiotics (such as Lactobacillus reuteri) and dietary fiber interventions, and providing a basis for developing GPR43 agonists or selective HDAC6 inhibitors (18); the circadian oscillation of its receptor further drives the clinical translation of chrono-nutritional strategies (such as timed fiber supplementation) (15). The bile acid/FXR/RUNX2-Wnt cascade validates the pharmacological effects of FXR agonists (such as geniposide) and microbiota-modulating agents on bone formation, opening up new avenues for OP drug development (11). The tryptophan metabolite-AhR axis promotes bone formation by repairing the intestinal barrier and activating the Wnt signal, supporting the application of AhR ligands, tryptophan-metabolizing probiotics, or functional nutrients to synergistically inhibit NLRP3 inflammasome activation (64). The bone-protective effects of H_2_S suggest that H_2_S donors (such as sodium hydrosulfide) or H_2_S-producing microbial transplantation may reduce oxidative stress damage in osteoblasts (105).

The regulation of the immune-metabolic network provides scientific support for probiotics, FMT, and cytokine-targeted therapies (such as anti-TNF-α biologics) to reshape the Th17/Treg balance and inhibit inflammatory bone loss. Especially crucial is the “metabolite-immune-circadian” triad model, which integrates circadian rhythms into therapeutic design and advocates for time-resolved intervention strategies based on host-microbe metabolic rhythms. The precise correspondence between these mechanistic targets and therapeutic approaches marks a substantive leap from basic discovery to clinical translation—offering a new paradigm for precision microbiota-based therapies for OP, OA, and other related diseases (**Table 1**).

**Table 1:** Key mechanisms, effects, and therapeutic strategies of gut microbiota-derived metabolites in skeletal disorders.

Metabolites	Signaling pathway	Key function/mechanism	Effects on bone cells	Direction of effect	Related diseases	Intervention strategies	Ref.
SCFAs	GPR43/FFAR2; HDAC inhibition	↓ osteoclastogenesis; promote osteoblast glycolysis shift; epigenetic modulation (e.g., H3K27ac); circadian synchronization	↓ osteoclastogenesis; promote osteoblast function	→	OP, OA	SCFA-producing probiotics (e.g., L. reuteri); dietary fiber; GPR43 agonists; HDAC6 inhibitors	(14, 17, 18, 40, 41)
Bile Acids	FXR; Wnt/β-catenin	↑ osteoblast differentiation and mineralization via RUNX2/Osx; ↓osteoclastogenesis; ↑ bone formation	↑ osteogenesis; ↓ osteoclast differentiation	→	OP, OA	FXR agonists (e.g., geniposidic acid - GPA); microbiota-modulating agents	(5, 48, 50, 51)
Tryptophan Derivatives	AhR; Wnt/β-catenin	Repair intestinal barrier; inhibit NLRP3 inflammasome; promote osteoblastogenesis; regulate osteoimmunity	↑ osteogenesis; ↓ osteoclasts	→	Inflammatory bone loss, OP	AhR ligands; tryptophan-metabolizing probiotics; functional nutrients	(64, 65)
Gut-derived Serotonin	–	↓ bone formation	↓ osteoblast proliferation	←	OP	–	(106, 107)
Brain-derivedSerotonin	–	↑ bone formation and inhibit bone resorption	↑ osteoblast activity; ↓ osteoclastogenesis	→	OP	–	(106, 107)
Melatonin	–	↑ osteoblast differentiation; anabolic and anti-resorptive effects	↑ osteogenesis; ↓ resorption	→	OP	–	(3, 108)
Kynurenine Pathway	–	↓ BMSC proliferation and osteoblast differentiation; promote bone aging	↓ bone formation	←	OP	–	(68, 69, 109)
Tryptophan Metabolites (IAA, IPA)	AhR; Wnt/β-catenin	Repair intestinal barrier; activate AHR; stimulate Wnt/β-catenin; promote osteogenesis	Promote osteogenesis; improve bone density	→	OP	Probiotics; tryptophan supplementation	(64–67)
H_2_S	Wnt/β-catenin; NF-κB; AMPK	Antioxidant; anti-apoptotic; ↑ osteoblast differentiation; inhibits inflammation-induced osteoclastogenesis	↑ osteogenesis; ↓ osteoclastogenesis	→	OP, OA	H_2_S donors (e.g., NaHS); transplantation of H_2_S-producing bacteria (e.g., Desulfovibrio)	(73–75, 105)
NO	cGMP-PKG pathway	Regulation of osteoclast-osteoblast coupling; ↑ bone defect repair	Bidirectional regulation	↔	Bone defects, OP	NO-releasing bone implant materials	(3, 73)
CO	HO-1 pathway	Anti-inflammatory; ↑ bone repair	↑ bone repair	→	BII, osteonecrosis	Targeting HO-1 pathway	(73)
NH_3_	–	↑ osteoclast activity; aggravates osteolysis	↑ osteoclast activity	←	Multiple myeloma	Targeting ammoniagenic bacteria	(73)
Immune- Metabolic Network	Th17/Treg; TNF-α,IL-1β; RANKL/OPG	Modulate systemic inflammation; regulate osteoclastogenesis; maintain bone-immune homeostasis	Modulate bone remodeling	Variable	OP, OA, RA	Probiotics; Prebiotics; FMT; Anti-cytokine biologics (e.g., anti-TNF-α, anti-IL-1β)	(49, 82–84, 87)
Circadian Integration	GPR43, FXR; H3K27ac	Synchronize osteoblast-osteoclast dynamics through epigenetic-immune coordination; temporal regulation of bone remodeling	Synchronize bone remodeling	→	OP, OA	Chronotherapeutic interventions; time-based probiotic/fiber dosing	(10, 17, 24)

↑, Promotes/increases; ↓, Inhibits/decreases; →, Positive; ←, Negative ↔, Bidirectional; AhR, Aryl Hydrocarbon Receptor; AMPK, AMP-activated Protein Kinase; BII, Bone implant infection;CO, Carbon Monoxide;FMT, Fecal Microbiota Transplantation; FXR, Farnesoid X Receptor; GPA, Geniposidic Acid; GPR, G Protein-Coupled Receptor; GPR43, G Protein-Coupled Receptor 43; HDAC, Histone Deacetylase; HO-1, Heme Oxygenase-1; H_2_S, Hydrogen Sulfide; IAA, Indole-3-Acetic Acid; IL, Interleukin; IPA, Indole-3-Propionic Acid; NaHS, Sodium Hydrosulfide; NF-κB, Nuclear Factor Kappa-Light-Chain-Enhancer of Activated B Cells; NH_3_, Ammonia; NLRP3, NLR Family Pyrin Domain Containing 3; NO, Nitric Oxide; OA, Osteoarthritis; OP, Osteoporosis; OPG, Osteoprotegerin; RA, Rheumatoid Arthritis; RANKL, Receptor Activator of Nuclear Factor Kappa-B Ligand; RUNX2, Runt-Related Transcription Factor 2; SCFAs, Short-Chain Fatty Acids; TNF-α, Tumor Necrosis Factor Alpha; Treg, Regulatory T Cell; Wnt, Wingless-related Integration Site.

### 3.5 Metabolite-bone temporal dynamics

The circadian oscillations of gut microbiota metabolites are a key bridge connecting the host circadian clock with bone metabolism. The production of SCFAs peaks during the active phase, in sync with the host’s feeding patterns. Studies have shown that the concentrations of acetate, propionate, and butyrate in the cecum of mice are 30-50% higher during the dark (active) phase than during the light phase (115). This rhythmic variation directly regulates the diurnal fluctuations of bone metabolism: SCFAs inhibit osteoclast differentiation at night through GPR43 signaling, while bile acids promote osteoblast differentiation during the day via FXR signaling, forming a complementary temporal coordination (48). The specific mechanisms involve multi-level spatiotemporal control: time-restricted feeding can enhance the circadian amplitude of SCFAs and improve bone density. In mouse models, restricting the feeding window to 12 hours within the active phase can increase bone formation rates by 25% (115). The circadian rhythm of glucocorticoids regulates the temporal distribution of metabolites by modulating gut permeability and microbial composition. The morning peak of cortisol can enhance SCFA production, indirectly promoting daytime bone formation (14, 95, 116). Individual circadian pattern variations significantly impact the function of the gut-bone axis. Polymorphisms in the PER3 gene lead to differences in “morningness” and “eveningness” chronotypes, affecting the phase of metabolite rhythms. Individuals carrying the long allele of PER3 have a 2–3 hour phase advance in SCFA rhythms, which may explain their differential susceptibility to shift work-related bone loss (117, 118). Age-related attenuation of rhythmic amplitude also leads to flattened metabolite oscillations in the elderly, which is associated with an increased risk of osteoporosis (62, 119).

## 4 Biomarkers in the gut-bone axis: implications for pathogenesis and clinical management

GBA represents a dynamic, bidirectional communication network between the gastrointestinal and skeletal systems, orchestrated through immune, metabolic, and microbiome-derived signaling pathways. Inflammatory biomarkers such as TNF-α and IL-6 play pivotal roles in promoting osteoclastogenesis via RANKL/NF-κB activation in inflammatory conditions like inflammatory bowel disease (IBD) and rheumatoid arthritis, while C-reactive protein (CRP) levels are inversely correlated with bone mineral density (87, 110). Meanwhile, bone turnover markers—including β-CTX (reflecting osteoclast activity) and PINP/osteocalcin (indicating osteoblast function)—are frequently dysregulated in metabolic bone diseases. Notably, gut microbiota influence osteocalcin’s γ-carboxylation, further linking microbial ecology to bone metabolism (111, 112). Together, these biomarkers facilitate early detection of inflammatory bone loss and allow for monitoring therapeutic responses to biological agents such as anti-TNF-α antibodies.

Gut microbiota-derived metabolites are central regulators of bone homeostasis. SCFAs exert osteoprotective effects by promoting regulatory T cell (Treg) differentiation and inhibiting osteoclastogenesis, whereas lipopolysaccharide (LPS) exacerbates bone resorption through TLR4/NF-κB signaling (113). These mechanisms are exemplified in germ-free mice, which exhibit elevated bone mass accompanied by reduced osteoclast precursors (CD11b+/Gr1-), lowered TNF-α and IL-1 expression, and altered serotonin metabolism due to suppressed Tph1 activity (120, 121). Additionally, hormonal regulators—including vitamin D (25(OH)D3), parathyroid hormone (PTH), and fibroblast growth factor 23 (FGF23)—modulate bone mineralization. Deficiencies in these factors often lead to secondary hyperparathyroidism and accelerated bone loss, particularly in malabsorption syndromes.

In OA, the bone-cartilage interface offers critical biomarker insights: CTX-II levels correlate with osteophyte formation (122, 123),and IL-1β drives cross-tissue degradation via matrix metalloproteinase activation (124). Proteomic studies further indicate dysregulation of subchondral bone during early OA, though these findings await broader validation. The RANKL/OPG ratio and zonulin—a marker of intestinal permeability—provide additional mechanistic insight, with elevated RANKL observed in postmenopausal osteoporosis and zonulin predicting bone loss associated with microbial translocation (114). Collectively, these findings support the use of combined biomarker panels incorporating gut-immune signals (e.g., serotonin, TNF-α) and osteochondral markers (e.g., CTX-II, IL-1β) for a more comprehensive assessment of bone health.

Clinically, integration of GBA biomarkers enables mechanistic stratification of bone disorders (e.g., inflammatory vs. metabolic origins) and guides personalized interventions such as microbiome modulation, nutritional support, and targeted biologics like anti-IL-1β agents in OA (125, 126). Future multi-omics approaches—including metagenomics and metabolomics—promise to unveil novel biomarkers, advancing precision medicine for osteoporosis, inflammatory osteopathies, and age-related skeletal decline. This integrative framework not only aids early risk detection in high-risk populations such as IBD patients but also enables monitoring of OA progression through osteochondral markers and supports the development of novel therapies targeting gut-microbiota-bone signaling pathways.

Recent single-cell transcriptomic studies have revealed circadian oscillations in metabolite receptors (e.g., GPR43, FXR) within bone marrow stromal cells. Disruption of these rhythms in osteoporosis patients underscores a “metabolite-immune clock” model, wherein microbial metabolites help synchronize osteoblast-osteoclast dynamics through epigenetic mechanisms such as H3K27ac acetylation (114) (**Table 2**) (142).

**Table 2:** Biomarkers of bone diseases in the gut-bone axis.

Category	Marker	Function	Key mechanism	Related diseases	Refs.
Inflammation	TNF-α	Pro-inflammatory; ↓OB; ↑OC	Activates NF-κB; ↑RANKL	RA, IBD, OP	(5, 20, 21, 87)
	IL-6	↑Osteoclastogenesis; ↓bone formation	Activates JAK/STAT	IBD, postmenopausal OP	(5, 20, 21, 87)
	IL-17	↑Bone resorption; ↓bone formation	Stimulates RANKL secretion	AS, PsA	(88)
	CRP	Systemic inflammation marker; ↓BMD	Negatively correlates with BMD	Chronic bone loss	(87, 110)
Bone Metabolism	β-CTX	Bone resorption marker	Collagen breakdown, OC activity↑	OP, Paget’s disease	(111)
	PINP	Bone formation marker	Collagen synthesis, OB activity ↑	OP, osteomalacia	(111)
	OC	Regulates mineralization; bone formation	Secreted by osteoblasts; reflects bone formation; Gut microbiota modulates γ-carboxylation	OP, fracture risk	(111, 112)
	TRAP-5b	OC-specific enzyme; bone resorption	↑Acid phosphatase activity	Multiple myeloma, OP	(111)
Gut Microbiota	SCFAs	Anti-inflammatory; ↓OC; ↑bone formation	Activate GPR43/41; ↑Treg differentiation	IBD, OP	(14, 17, 18, 113)
	LPS	Pro-inflammatory; ↑bone resorption	Activates TLR4/NF-κB; ↑RANKL	Metabolic bone disease	(25, 26, 37, 86)
Vitamins/ Hormones	25(OH)D3	↑ Ca²^+^ absorption; bone mineralization	Deficiency → secondary hyperparathyroidism	Rickets, OP	(5, 8, 14)
	PTH	Regulates Ca²^+^/P metabolism; ↑bone resorption	↑OC activation in hypocalcemia/VitD deficiency	Secondary OP, renal osteodystrophy	(5)
	FGF23	↓Phosphate reabsorption; modulates VitD	Gut dysbiosis may alter expression	CKD-MBD	(5)
Other Molecules	RANKL/OPG	RANKL: ↑OC; OPG: inhibitor	Gut inflammation ↑RANKL; disrupts balance	Postmenopausal OP, RA	(57, 58, 114)
	Zonulin	Gut permeability marker	Barrier breach → LPS translocation → bone loss	CD, IBD-related OP	(9, 114)
	IgA	Gut immune regulation	Mucosal immunity disruption effects bone metabolism	Autoimmune bone disorders	(5, 49)

↑, Promotes/increases; ↓, Inhibits/decreases; AS, Ankylosing Spondylitis; BMD, Bone Mineral Density; CKD-MBD, Chronic Kidney Disease-Mineral and Bone Disorder; CD, Celiac disease; CRP, C-Reactive Protein; FGF23, Fibroblast Growth Factor 23; GPR, G Protein-Coupled Receptor; IBD, Inflammatory Bowel Disease; IgA, Immunoglobulin A; IL, Interleukin; LPS, Lipopolysaccharide; NF-κB, Nuclear Factor Kappa-Light-Chain-Enhancer of Activated B Cells; OB, Osteoblast; OC, Osteocalcin; OP, Osteoporosis; OPG, Osteoprotegerin; PINP, Procollagen Type I N-Terminal Propeptide; PsA, Psoriatic Arthritis; PTH, Parathyroid Hormone; RA, Rheumatoid Arthritis; RANKL, Receptor Activator of Nuclear Factor Kappa-B Ligand; SCFAs, Short-Chain Fatty Acids; TLR4, Toll-Like Receptor 4; TNF-α, Tumor Necrosis Factor Alpha; TRAP-5b, Tartrate-Resistant Acid Phosphatase 5b; Treg, Regulatory T Cell; VitD, Vitamin D.

## 5 Clinical interventions of bone disorders based-GBA

### 5.1 Dietary interventions

The gut microbiota significantly influences bone health through nutrient-mediated mechanisms, particularly by enhancing monosaccharide absorption and energy harvesting (5, 23, 140, 143). Specific strains, such as Lactobacillus plantarum, have been shown to stimulate skeletal development under malnutrition conditions, suggesting that microbiota modulation could optimize nutrient utilization for bone health enhancement. Dietary fiber bridges gut microbiota and bone health through microbial fermentation-derived SCFAs. This process not only acidifies the intestinal environment but also generates SCFAs that reinforce gut barrier integrity and suppress systemic inflammation, thereby indirectly supporting skeletal homeostasis (127, 128). Increased dietary fiber intake positively modulates microbial composition, establishing a beneficial dietary-microbiota-bone axis (144, 145).

### 5.2 Prebiotics

Prebiotics, including inulin and fructooligosaccharides (FOS/GOS), augment mineral bioavailability through gut microbiota remodeling. Fermentation of these fibers elevates SCFAs production, which acidifies intestinal pH to enhance calcium solubility and activates mineral transport pathways (129). Clinical trials demonstrate that daily intake of 8 g inulin-type fructans increases calcium absorption by 12% in adolescents and improves whole-body bone mineral density within one year, paralleled by Bifidobacterium enrichment (129, 130). Similarly, soluble corn fiber enhances calcium retention in postmenopausal women dose-dependently, correlating with increased Lachnospiraceae and Bacteroides abundance (130). Mechanistically, prebiotics reshape microbial composition (e.g., reduced Firmicutes/Bacteroidetes ratio) and modulate metabolic pathways for carbohydrate/lipid utilization, synergistically promoting calcium absorption and bone mechanical strength (130). These effects are further amplified by SCFA-mediated expansion of intestinal absorptive surface and permeability (130, 146). Collectively, prebiotic-driven microbiota modulation offers a dietary strategy to combat OP, particularly in calcium-deficient populations (129).

### 5.3 Probiotics

Probiotics demonstrate osteoprotective effects through microbiota-immune interactions. Lactobacillus reuteri enhances bone density in healthy male mice by suppressing intestinal inflammation and restructures gut microbial communities in ovariectomy (Ovx) models, effectively attenuating estrogen deficiency-induced bone loss (131, 147). Mechanistically, L. reuteri inhibits osteoclastogenesis via dual pathways: downregulating bone marrow CD4+ T cell recruitment (key osteoclast activators) and directly suppressing osteoclast differentiation *in vitro*. These actions correlate with reduced bone resorption markers and preserved trabecular architecture (131, 148). Similarly, L. paracasei and L. plantarum mitigate hormone deficiency-driven OP, highlighting genus-specific modulation of bone remodeling pathways (130, 148–150).

### 5.4 FMT

The efficacy of FMT in treating OP exhibits considerable heterogeneity, influenced by factors including donor selection (e.g., potential fecal pathogens affecting safety) (137, 138), recipient response, host gut microbiota composition/function, donor microbial diversity, immune status, and administration protocols (151, 152). Variations in donor microbiota, host immunity, and delivery methods further contribute to inconsistent outcomes (153). The composition and function of the donor microbiota are the “cornerstones” determining the success of FMT. The concept of “super donors” suggests that certain donors’ microbiota have higher engraftment capabilities and stability. For example, the presence of Akkermansia muciniphila (which improves gut barrier function) (154), Faecalibacterium prausnitzii (anti-inflammatory) (155), and high SCFA-producing Lachnospiraceae and Ruminococcaceae in the donor microbiota are associated with better osteoprotective effects (23). Conversely, if potential pathogens (such as Enterobacteriaceae) in the donor microbiota are not adequately screened, it may lead to FMT failure or even exacerbate bone loss. Additionally, the recipient’s intrinsic environment serves as the “soil” for microbial engraftment, and its condition is crucial. The recipient’s low microbial diversity, high proportion of antibiotic-resistant bacteria, or specific microbial structure can resist the implantation of new microbiota, a phenomenon known as “colonization resistance.” For instance, a high abundance of Bacteroides in the recipient’s gut may hinder the engraftment of other strains. The host’s immune status (such as Treg/Th17 balance), genetic background (such as polymorphisms in immune-related genes), and gut barrier integrity together form a unique microenvironment that determines the fate of the transplanted microbiota and the variability of immune modulation effects (156). Moreover, the type and preparation of the FMT formulation also affect the efficacy. Different methods, such as fresh versus frozen fecal preparations and oral capsules versus endoscopic infusion, have significant impacts on microbial viability and delivery efficiency. The freeze-thaw process may damage the viability of some strictly anaerobic bacteria, while the enteric coating technology of capsules affects the precise release of microbiota in the gut.

FMT restores gut microbiota diversity and shows osteoprotective effects in preclinical models. In ovariectomy-induced OP, FMT inhibits osteoclastogenesis and improves bone density via microbiota-metabolite interactions; in OA, it reduces inflammation through Th17/Treg modulation (52, 132). Clinical data indicate immunomodulatory shifts in IBD patients after FMT, suggesting bone benefits via the gut-bone axis (149, 157). Mechanisms include enrichment of anti-inflammatory taxa, regulation of bone remodeling via microbial metabolites (e.g., SCFAs), and modulation of osteoclast-related cytokines (149, 158).

Future directions involve elucidating microbiota-bone crosstalk, validating efficacy via RCTs, and exploring combination therapies with nutritional or pharmacological interventions. Preventive FMT applications in high-risk populations warrant further investigation (**Supplementary Table 1**).

### 5.5 Traditional Chinese medicine

TCM represents a holistic medical system with a history spanning thousands of years. For the purpose of this review, which focuses on the GBA and metabolic regulation, we specifically refer to the phytochemical or herbal medicine component of TCM, rather than practices such as acupuncture or Tai Chi. Numerous active compounds derived from TCM herbs have demonstrated potent osteogenic, angiogenic, and anti-inflammatory properties in preclinical studies, positioning them as promising candidates for bone tissue engineering and the management of metabolic bone diseases. Among the most extensively studied osteo-promotive TCM compounds are flavonoids (e.g., icariin and icaritin) (159), polyphenols (e.g., resveratrol) (160, 161). To enhance clarity for a global readership, we will elucidate the botanical origins and mechanisms of these specific compounds:

Icariin and Icaritin: These are prenylated flavonoids isolated from the herb *Epimedium* (commonly known as Horny Goat Weed). They are recognized as principal active constituents responsible for the bone-strengthening effects traditionally attributed to *Epimedium* in TCM theory. Mechanistically, icariin and icaritin significantly enhance the proliferation and differentiation of osteoblasts by upregulating key osteogenic genes, including RUNX2, ALP, Osteocalcin (OCN), and Collagen I (COLI) (159, 162). Furthermore, they promote intraosseous angiogenesis—a critical process for bone regeneration—via the VEGF and HIF-1α pathways (56, 133–136, 139, 141, 163–168).

Resveratrol: While also present in grapes and berries, resveratrol is a stilbenoid polyphenol abundantly found in *Polygonum cuspidatum* (Japanese Knotweed), a plant used in TCM. Similar to baicalin, resveratrol exhibits strong anti-inflammatory and antioxidant effects (160, 161). It helps protect bone cells from oxidative damage and suppresses pro-inflammatory pathways, thereby preserving bone mass and promoting a healthy remodeling balance.

Additionally, TCM components like baicalin and resveratrol exhibit potent anti-inflammatory and antioxidant effects, mitigating oxidative stress and creating a favorable microenvironment for bone regeneration (136, 167–169).

**Figure 6:**
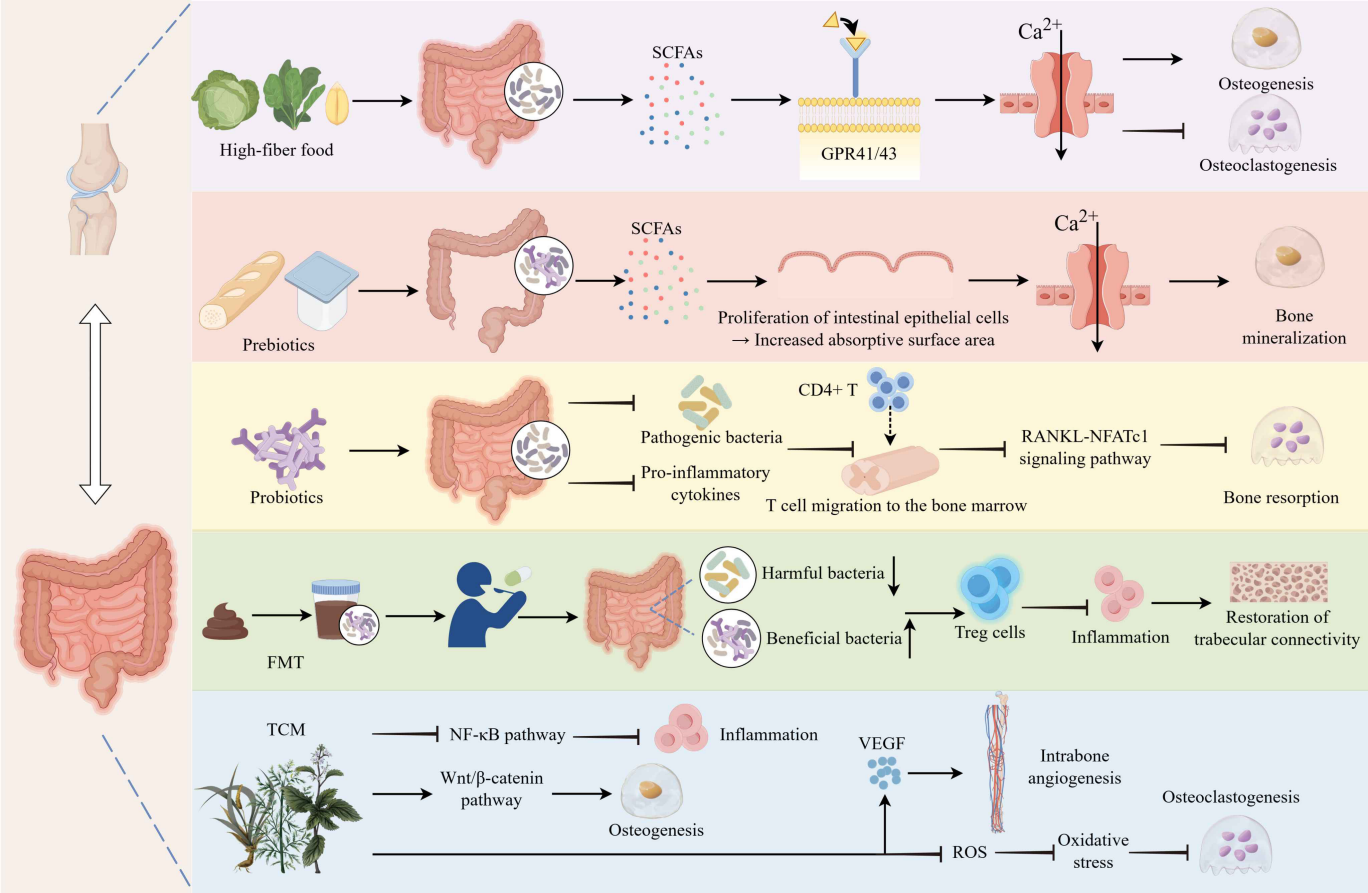
Five-pronged therapeutic strategies targeting the gut-bone axis to restore skeletal homeostasis. ① High-fiber diets yield microbiota-derived short-chain fatty acids (SCFAs) that activate G Protein-Coupled Receptor (GPR)41/43, enhancing calcium absorption and osteogenesis while suppressing osteoclastogenesis. ② Prebiotics (e.g., inulin) amplify SCFA production, promoting intestinal epithelial proliferation to increase absorptive surface area and expand beneficial bacteria.③ Probiotics (e.g., *Lactobacillus*) directly inhibit pathogenic bacteria, reduce pro-inflammatory cytokines (TNF-α/IL-6), and block RANKL-NFATc1 signaling to limit T-cell migration to bone marrow and bone resorption. ④ Fecal microbiota transplantation (FMT) restores microbial balance by elevating beneficial taxa and suppressing harmful bacteria, augmenting Treg cells to resolve inflammation-driven bone loss. ⑤ Traditional Chinese Medicine (TCM) activates the Wnt/β-catenin pathway and VEGF-mediated intra-bone angiogenesis to stimulate osteogenesis, while inhibiting NF-κB and reactive oxygen species (ROS) to mitigate oxidative stress and resorption. These interventions collectively remodel the bone microenvironment through immunometabolic crosstalk.↑: Promotes/increases; ⊥: Inhibits/decreases.

While the direct modulation of the microbiota-GBA by TCM remains underexplored, its systemic effects on bone metabolism and inflammation suggest potential interactions with gut microbiota. For example, TCM may enhance gut barrier function and reduce systemic inflammation by modulating microbial composition and metabolite production, such as SCFAs and bile acids, thereby indirectly supporting bone health (167, 170). Future research should focus on elucidating these mechanisms to optimize TCM-based strategies for bone regeneration and disease management (**Figure 6**) (**Table 3**).

**Table 3:** Systematic comparison of clinical interventions based on the gut-bone axis.

Category	Dietary interventions	Probiotics	Prebiotics	FMT	TCM	Refs.
Mechanism of Action	Boost gut barrier; ↓inflammation	Modulate microbiota; ↓osteoclast differentiation	↑SCFAs; ↑Ca^2+^ absorption	Restore microbiota balance; ↓LPS/TLR4 → ↓inflammation	Multi-target (e.g., Icariin→Wnt/β-catenin); Antioxidant	(52, 78, 79, 127–136)
Clinical Efficacy	12%↑BMD (teens, inulin); 12%↑Ca^2+^ absorption	5–10%↑BMD (postmenopausal); 25%↓IL-6	8%↑BMD (postmenopausal, inulin); 15%↑Ca^2+^ retention	15%↑BMD (OVX mice); 32%↓TNF-α (RCT)	8–12%↑BMD; 20%↑osteocalcin	(52, 84, 129–132, 135, 136)
Type of Study and Level of Evidence	RCT (B)	RCT Phase II (B)	RCT + Animal (B)	Animal + RCT Phase II (B)	Meta-analysis + Cellular (C)	(52, 84, 129, 130, 132) (135, 136)
Advantages	Safe, low cost, good adherence	Strain-specific efficacy; combinable	Broad applicability (no live bacteria)	Potent (refractory cases); holistic	Multi-component synergy; high acceptance	(52, 78, 127, 129, 131–134)
Limitations	Slow effect, limited in severe cases	Strain variability; unstable long-term effects	High dose → GI discomfort	Unknown long-term safety; donor screening	Complex composition; lack of RCTs	(127, 130, 131, 137–139)
Target Population	Adolescents/poor Ca^2+^ absorption; early OP prevention	Postmenopausal women; mild OP	Elderly/inadequate Ca^2+^ intake; high-risk OP	Severe OP; arthritis w/bone destruction	Chronic bone diseases; natural therapy preference	(52, 84, 129, 130, 132, 135, 136)
Ethics and Compliance	No ethics approval; low privacy risk	GRAS; strain safety assessment	GRAS; dose standardization	IRB approval; donor anonymization (GDPR)	Minimal ethical issues; GMP required	(78, 127, 129, 137, 139)
Future Research Directions	Personalized diets; long-term BMD tracking	Strain-metabolite combos; dose optimization	New prebiotic screening; multi-population validation	Freeze-dried capsules; 10-yr safety follow-up	Monomer mechanism (e.g., icariin); multicenter RCTs	(131, 135, 137, 140, 141)

↑, Increase/improvement; ↓, Decrease/reduction; BMD, Bone Mineral Density; Ca, Calcium; FMT, Fecal Microbiota Transplantation; GDPR, General Data Protection Regulation; GI, Gastrointestinal; GMP, Good Manufacturing Practice; GRAS, Generally Recognized As Safe; IRB, Institutional Review Board; OP, Osteoporosis; OVX, Ovariectomized; RCT, Randomized Controlled Trial; SCFA, Short-Chain Fatty Acid; TCM, Traditional Chinese Medicine.

## 6 Challenges and translational prospects

Microbiota-bone axis research is still hampered by three major gaps: germ-free models fail to replicate the complexity of the human microbiota, individual metabolite receptor circadian peak phases differ by 4–6 hours, and FMT colonization is highly heterogeneous. Existing clinical evidence supports the immediate implementation of “chronotherapy” for skeletal health: taking bisphosphonates on an empty stomach in the morning increases bioavailability by 20–30%, calcium supplementation in the evening can additionally reduce nocturnal CTX by 15%, taking vitamin D with a fat-containing meal at noon is in phase with the solar rhythm, and orally administering SCFA-producing Lactobacillus reuteri in the morning increases lumbar bone density by 40% in postmenopausal women. Shift work and jet lag increase the risk of hip fractures by 32% through three pathways: reduced microbial rhythm amplitude with a phase delay of 4–6 hours, increased intestinal LPS translocation, and sustained elevation of IL-6 and TNF-α. Corresponding interventions include avoiding high-fat diets during night shifts and supplementing with resistant starch during the “biological morning” to partially restore SCFA rhythms and inhibit bone resorption. The next step is to develop CRISPR-engineered strains that continuously secrete butyrate and FXR agonists, and combine them with an AI-chronotherapy platform to analyze individual 24-hour single-cell transcriptome and plasma metabolome curves, generating a triphasic chronotherapy schedule for “drugs-nutrition-microbiota,” which will be included in Phase II randomized controlled trials to further enhance bone density benefits by one effect size.

## 7 Conclusion

This review elucidates how gut microbiota-derived metabolites (e.g., SCFAs, bile acids, and tryptophan derivatives) regulate bone homeostasis through a novel “metabolite–immune–bone triad” model, incorporating circadian biology to reveal rhythmic expression of receptors (e.g., GPR43, FXR) in bone stromal cells and their synchronization of osteoblastic/osteoclastic activities via epigenetic mechanisms such as H3K27ac acetylation. These insights deepen our understanding of the pathogenesis of OP and OA and provide a mechanistic foundation for targeting the GBA through precision interventions including chrononutrition, probiotics, and microbiota transplantation. Our work underscores the transformative potential of multi-omics-guided approaches and individualized temporal therapeutics for future research and clinical translation in skeletal disorders.
