# Supplementary material for: Multi-omics reveals circadian regulation of bone homeostasis by gut microbiota metabolites: mechanisms and chronotherapeutic implications
Source: Front Immunol. 2026 May 28;16:1719445. doi: 10.3389/fimmu.2025.1719445 (PMC13260651; doi:10.3389/fimmu.2025.1719445)
Supplement: Supplementary file 1 [file Table1.docx]

**Supplement Table 1. **Summary of Efficacy Heterogeneity, Mechanisms, and Clinical Application Prospects of FMT in the Treatment of Osteoporosis****

| **Category** | **Factors/Aspects** | **Specific Description** |
| --- | --- | --- |
| **Heterogeneity Factors** | Donor Selection | Pathogens in donor feces may affect safety and efficacy. |
|  | Recipient Response & Host Status | Host gut microbiota composition/function and immune status can impact efficacy. |
|  | Administration Protocols | FMT delivery methods and dosage can cause efficacy differences. |
|  | Microbiota Alterations | Specific bacteria may mitigate bone loss, while others may worsen conditions after FMT. |
|  | p53 Deficiency | p53 deficiency may disrupt lactate metabolism, impairing FMT efficacy. |
| **Efficacy Evidence** | Diversity Restoration | Treatment success for recurrent *C. difficile* infection reaches 90% in observational studies. |
|  | Preclinical - OP | FMT improves bone density and structure in Ovx-induced OP models. |
|  | Preclinical - OA | FMT reduces joint inflammation in osteoarthritis models by modulating Th17/Treg balance. |
|  | Clinical Potential - IBD | Microbiota remodeling in IBD patients suggests skeletal benefits via the GBA. |
| **Mechanisms** | Microbial Reshaping | Remodel microbiota to enrich anti-inflammatory taxa. |
|  | Metabolite Signaling | Regulate osteoblast-osteoclast balance. |
|  | Immune Modulation | Target osteoclastogenic factors through systemic immune modulation. |
| **Future Directions** | Mechanistic Studies | Clarify microbiota-bone crosstalk pathways. |
|  | Clinical Validation | Validate FMT efficacy in RCTs for osteoporosis/osteoarthritis. |
|  | Combinatorial Therapies | Explore combination therapies. |
|  | Preventive Applications | Investigate FMT's preventive use in high-risk populations. |

****Key Abbreviations: FMT****: Fecal Microbiota Transplantation; **GBA**: Gut-Bone Axis; **IBD**: Inflammatory Bowel Disease; **OP**: Osteoporosis; **Ovx**: Ovariectomized; **RCT**: Randomized Controlled Trial.
